# *Cor*relating tumor metabolic progression *i*ndex measured by serial FDG PET-CT, apparent diffusion coefficient measured by magnetic resonance imaging (MRI) and blood genomics to patient’s *o*utcome in advanced co*l*orectal c*an*cer: the CORIOLAN study

**DOI:** 10.1186/1471-2407-14-385

**Published:** 2014-05-30

**Authors:** Amelie Deleporte, Marianne Paesmans, Camilo Garcia, Caroline Vandeputte, Marc Lemort, Jean-Luc Engelholm, Frederic Hoerner, Philippe Aftimos, Ahmad Awada, Nicolas Charette, Godelieve Machiels, Martine Piccart, Patrick Flamen, Alain Hendlisz

**Affiliations:** 1 Medicine Department, Institut Jules Bordet, Université Libre de Bruxelles, Brussels, Belgium; 2 Data Centre, Institut Jules Bordet, Université Libre de Bruxelles, Brussels, Belgium; 3 Nuclear Medicine Department, Institut Jules Bordet, Université Libre de Bruxelles, Brussels, Belgium; 4 Radiology Department, Institut Jules Bordet, Université Libre de Bruxelles, Brussels, Belgium

**Keywords:** Colorectal cancer, Progression rate assessment, FDG-PET, PET/CT

## Abstract

**Background:**

Metastatic colorectal cancer (mCRC) may present various behaviours that define different courses of tumor evolution. There is presently no available tool designed to assess tumor aggressiveness, despite the fact that this is considered to have a major impact on patient outcome.

**Methods/Design:**

CORIOLAN is a single-arm prospective interventional non-therapeutic study aiming mainly to assess the natural tumor metabolic progression index (TMPI) measured by serial FDG PET-CT without any intercurrent antitumor therapy as a prognostic factor for overall survival (OS) in patients with mCRC.

Secondary objectives of the study aim to test the TMPI as a prognostic marker for progression-free survival (PFS), to assess the prognostic value of baseline tumor FDG uptake on PFS and OS, to compare TMPI to classical clinico-biological assessment of prognosis, and to test the prognostic value on OS and PFS of MRI-based apparent diffusion coefficient (ADC) and variation of vADC using voxel-based diffusion maps.

Additionally, this study intends to identify genomic and epigenetic factors that correlate with progression of tumors and the OS of patients with mCRC. Consequently, this analysis will provide information about the signaling pathways that determine the natural and therapy-free course of the disease. Finally, it would be of great interest to investigate whether in a population of patients with mCRC, for which at present no known effective therapy is available, tumor aggressiveness is related to elevated levels of circulating tumor cells (CTCs) and to patient outcome.

**Discussion:**

Tumor aggressiveness is one of the major determinants of patient outcome in advanced disease. Despite its importance, supported by findings reported in the literature of extreme outcomes for patients with mCRC treated with chemotherapy, no objective tool allows clinicians to base treatment decisions on this factor. The CORIOLAN study will characterize TMPI using FDG-PET-based metabolic imaging of patients with chemorefractory mCRC during a period of time without treatment. Results will be correlated to other assessment tools like DW-MRI, CTCs and circulating DNA, with the aim to provide usable tools in daily practice and in clinical studies in the future.

**Clinical trials.gov number:**

NCT01591590.

## Background

### Natural history of metastatic colorectal cancer

With an incidence rate of 35 per 100.000 per year, colorectal cancer (CRC) affects about 150.000 people each year in Western Europe. Although surgery is a potentially curative treatment, about half of patients experience metastatic spread of their disease [1], which, in the vast majority of cases, leads to their death. Current management algorithms in mCRC are based on anatomical considerations defining the resectability of tumor spread, or clinical symptoms (ECOG general status, number of metastatic sites, alkaline phosphatase levels, transaminase levels).Clinical symptoms, however, provide only a partial picture of the situation. To date, the analysis of tumor biology, with the noticeable exception of RAS mutations, which are of interest only for anti-EGFR therapies, remains completely absent from most decision-making about mCRC.

The natural history of mCRC tumors has been poorly studied. However, a thorough review of the scientific literature highlights its importance. Six prospective, randomized trials involving chemotherapy-free intervals in at least one of the randomization arms [2-8] have been published, and have enrolled 1149 patients whose treatment included a therapeutic temporary delay until progression. These trials can be classified into two types:

1) Studies comparing immediate versus delayed chemotherapy in first-line mCRC, and

2) Studies comparing chemotherapy-free intervals until clinical or radiological evidence of progression versus chemotherapy maintenance in patients having experienced disease control after 2 or 3 months of induction therapy.

Trials using first-line chemotherapy [3,5,7] report that 6% to 15% of tumors progress during the 2 to 3 months induction period, suggesting that these tumors most probably have a chemo-refractory and an aggressive phenotype.

By contrast, patients included in early trials at a time when only 5*-*fluorouracil was available are reported to have a median overall survival (OS) of 10 months. Interestingly, 8% to 19% of them are still alive after 2 years [2,4]. It is hypothesized that these patients bear slow-growing diseases that are probably partially sensitive to chemotherapy.

Progression-free-survival (PFS) of patients with tumors observed in a therapeutic window is usually measured at 3 to 6 months with large ranges from 0.1 to 30 months. Those large ranges prefigure the differences between several tumor subpopulations.

Moreover, two of the studies [3,5] show no correlation between length of CFI and subsequent response to chemotherapy, adding another indirect argument to support the hypothesis that tumor’s natural evolution and its sensitivity to chemotherapy mirror different aspects of the tumor.

Formal study of the natural pace of tumor evolution by classical means is difficult and, while additional evidence is obviously needed, new tools able to discriminate different paces of tumor growth must still be developed and validated.

### Assessment of tumor metabolic progression index (TMPI)

The clinical evidence for tumor aggressiveness has never been formally assessed in daily practice or in clinical studies and remains largely unpredictable. In both contexts, the patient populations are composed of a wide array of different tumor phenotypes evolving with different outcomes while carrying the same apparent disease.

Tailoring treatment to the tumor aggressiveness requires an objective and rapidly available mean to assess a tumor’s behavior. One could hypothesize that the same tools used to assess tumor response under therapy could also be used to assess natural tumor growth independently of the treatment given, for instance during a rest period. The most frequently used RECIST-based radiological response assessment has a definite but very limited descriptive value of treatment benefit in cancer care [9-13]. New biological drugs constitute an even greater challenge for classical radiology because they seldom induce structural changes to the tumor, underscoring the need to develop new diagnostic means to assess early drug-induced intra-tumoral changes. Such new assessment methods could lead to new trial designs based on intra-patient comparisons, circumventing patient and tumor heterogeneity.

Several potential early response detection techniques are emerging: serial FDG PET-CT; dynamic contrast-enhanced MRI (DCE-MRI) and diffusion MR; and circulating tumor cells (CTCs) and circulating tumor DNA [14] detection. Among these, FDG PET-CT is the most studied and has been found to be very promising. Its value in detecting early metabolic changes, predictive of a therapy’s later outcome, is currently widely assessed [15,16]. Recent data suggest that serial FDG PET-CT tumor metabolic assessment is a reliable tool for early detection of refractory disease, provided some conditions are fulfilled (e.g., tumor must be FDG-avid and lesions should be greater than a defined minimal size).

Higashi et al.’s trials on ovarian cancer cell lines suggest that FDG uptake does not relate to the proliferative activity of cancer cells, but strongly relates to the number of viable tumor cells [17]. If we know that the average doubling of mCRC cells is about 92 days [18], and if we accept that over time both cell volume and cellular glycolytic activity increase while the interstitial volume remains constant, then whole tumor FDG uptake should be linearly correlated with the number of cells. Moreover, it is important to detect tumors in their exponential growth period (rather than linear growth), given that for PET detectability there should be a minimal increase of 15% in SUVmax to be significant; in this way, a 2-week interval between two FDG PET-CT scans should be sufficient.

Previously, our research group prospectively included 42 patients with mCRC undergoing first- or second-line chemotherapy in a study investigating serial FDG PET-CT. FDG PET-CT was performed at baseline and 15 days after the first cycle of chemotherapy. Data show excellent correlation between the absence of metabolic response at day 14 and the absence of structural response as measured by CT Scan at 6 weeks, a modest correlation between metabolic and radiological response, and excellent predictive value for metabolic response on PFS and overall survival (OS) [19].

### FDG PET-CT assessments

Some groups have performed serial FDG PET-CT imaging without intercurrent treatment in cancer patients [20]. However, the aim of these studies was to determine the cut-off for defining a significant metabolic response or progression. The calculated variability in these studies was probably contaminated by the inclusion of rapidly progressing tumors that showed rapid FDG uptake increases, which were falsely considered to reflect measurement variability.

The variability of tumor FDG uptake measurement performed after 2 weeks without any antitumor drug interventions depends on several factors including 1) the variability of the measure for technical reasons, 2) the patient’s physiological conditions variations (e.g., insulin levels, fluctuations in tumor blood flow) and 3) TMPI. For the present study, it is of crucial importance that the first two sources of variability are minimized using very strict standardization of imaging.

The “technical” variability was found to be minimal in lesions bigger than 2 cm and lesions with high FDG uptake (high SUV).

### Magnetic resonance imaging

Diffusion-weighted magnetic resonance imaging (DW-MRI) is a technique used to reflect the microstructural properties of tissues, related to the intra- and extra-cellular motion of free water molecules, indicative of tissue cellularity and structure. Measurement and quantification are possible using the apparent diffusion coefficient (ADC) of DW-MRI and have been linked to lesion aggressiveness and tumor response, although the biophysical basis for this is not completely understood. Hyper-cellularity and increased nucleo-cytoplasmic ratio decrease ADC. Necrosis and loss of cells tend to increase ADC values. Parameters derived from DW-MRI are appealing as imaging biomarkers, because their acquisition is noninvasive. Moreover, DW-MRI does not require any exogenous contrast agents, does not use ionizing radiation, and yet results are quantitative and can be obtained relatively rapidly, being easily incorporated into routine patient evaluations.

Changes in DW-MRI may be an effective early biomarker for treatment outcome both for vascular disruptive drugs and for therapies that induce apoptosis [21,22]. Successful treatment is reflected by increases in ADC values. DW-MRI has also been shown to prospectively predict the success of some treatments in a number of different tumors [23-25]. Recently, Morgan et al. showed the potential of ADC variation over time to predict the natural history of untreated prostate cancer [26].

Acquisition sequences for DWI are not completely standardized, but basic techniques are well known and available on systems from all major vendors. There is no established standard for measurement of ADC but recent reports promote voxel-based analysis and volumetric evaluation of ADC (vADC) which is well correlated with cellularity, as shown in gliomas [27,28]. This method also carries the advantages of being less operator-dependent and more reproducible than ROI-based techniques. For a monocentric study, the ADC calculation is reproducible and robust over time. Longitudinal voxel-based measurements seem well suited to treatment follow-up.

### Next generation sequencing

Numerous studies have shown that the concentration of circulating cell-free tumor DNA is higher in cancer patients than in healthy individuals. Tumor cells release naked DNA into the plasma after apoptosis or necrosis, early in their development. Because this DNA can be extracted from blood, the measurement of circulating free DNA could be a potential new tool for cancer detection [14]. Moreover, the extracted DNA could be used to detect genetic and epigenetic alterations through Next Generation Sequencing (NGS) technologies that may affect the important regulatory pathways in the pathology of cancer.

Evaluating blood samples for mutant DNA is particularly attractive, because it could be applicable in diverse forms of cancer, including solid tumors, and because blood samples could easily be collected during the clinical follow-up of patients [29,30]. If one could show that specific genomic rearrangements in plasma DNA provide a sensitive and specific measure of tumor growth rate and that they can be used as an early biomarker of disease prognosis and patient outcome, this may provide a substantial advance in monitoring the disease burden in patients with CRC. In a trial enrolling 30 metastatic breast cancer patients, circulating tumor DNA provided the earliest measure of treatment response in 10 of 19 women (53%) when compared to CA 15–3 levels and the number of circulating tumor cells (CTCs) measured at the identical time point [31]. This technology appears very promising for studying the clonal evolution of metastatic cancer under therapy or during CFIs.

### Assessement of circulating tumor cells

CTCs are cells that originate from a primary tumor and circulate through the bloodstream. The FDA-approved CellSearch® system enables CTC enrichment by using antibody-coated magnetic beads. Previous studies have shown that CTCs, which can be detected and analyzed in a standardized, objective manner, may have prognostic and predictive value in the metastatic cancer setting, including metastatic breast [32,33] and colon cancer [34-36]. It would be interesting to validate whether CTC detection and quantification could serve as a clinically relevant surrogate marker of tumor growth or aggressiveness for the individual patient with mCRC.

### Study hypothesis

We hypothesize that, in a population of patients with mCRC for whom no known effective therapy is available, tumor growth rate is related to patient outcome, and that serial FDG PET-CT will be able to measure it. If the hypothesis is verified, this finding could enable us to define therapeutic strategies according to the TMPI assessed by serial pre-therapeutic FDG-PET. It would also limit the need for randomization in early drug development phases, because patients could be considered as their own control. Moreover, patients could be stratified according to their baseline metabolic growth rates in randomized controlled trials having OS as endpoint.

## Methods

### Study design

**Figure 1:**
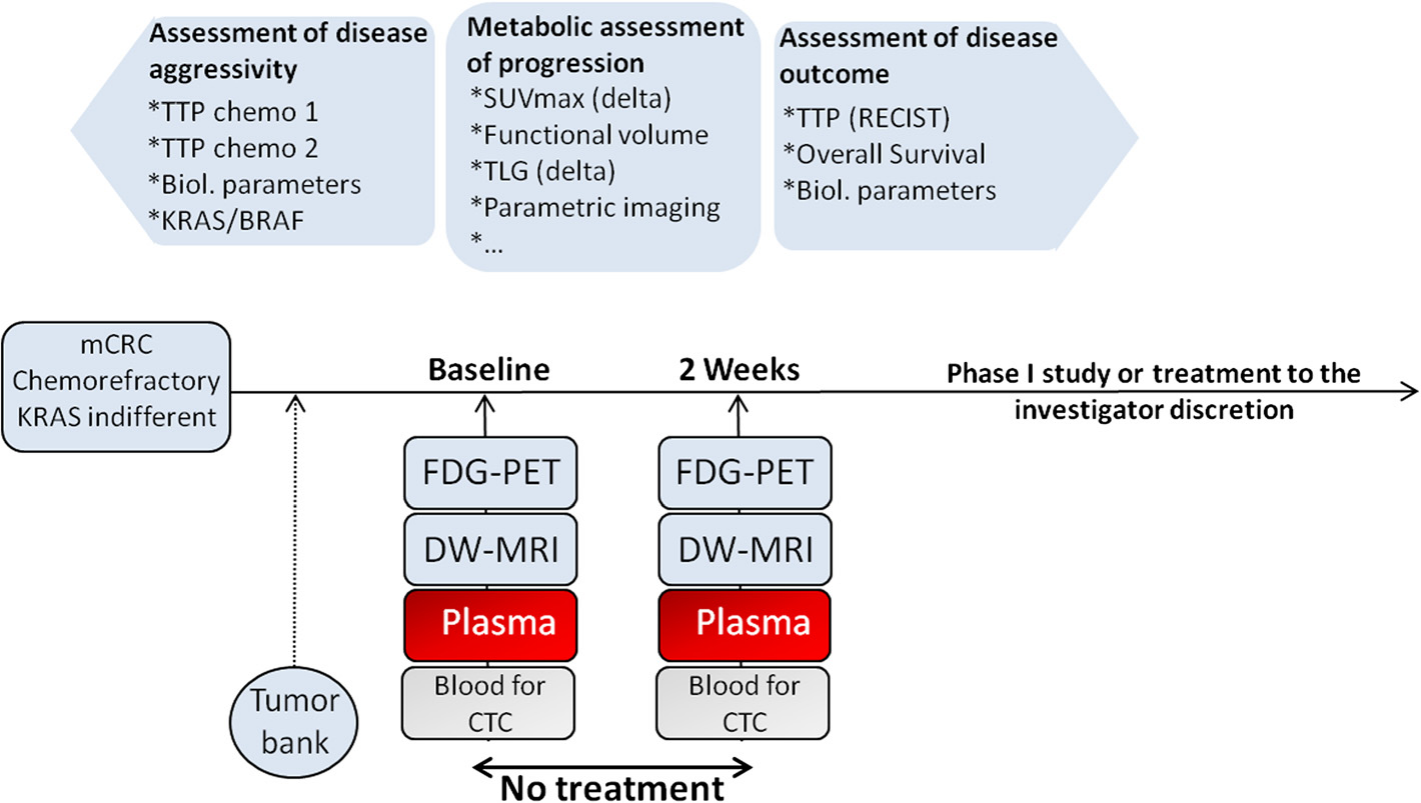
**Study design.** TTP = time to progression, SUV = Standardized Uptake Value; TLG = Total Lesion Glycolysis, mCRC = metastatic ColoRectal Cancer, FDG-PET: FluoroDeoxyGlucose-Positron Emission Tomography, DW-MRI = Diffusion-Weighted Magnetic Resonance Imaging, CTC: Circulating Tumor Cells).

The study is designed as a single-arm, prospective, interventional, non-therapeutic study to assess the value of FDG PET-CT in defining tumor metabolic progression in patients with mCRC during a period without treatment (see Figure 1 for an overview of the study design).

### Objectives

The primary objective of the study is to assess the spontaneous TMPI measured by serial FDG PET-CT without any intercurrent antitumor therapy as a prognostic factor for OS in patients with mCRC.

Secondary objectives are 1) to test TMPI as a prognostic marker for PFS; 2) to assess the prognostic value of baseline tumor FDG uptake on PFS and OS; 3) to compare TMPI to classical clinico-biological assessment of prognosis; and 4) to test the prognostic value of MRI-based apparent diffusion coefficient (ADC) and variation of vADC using voxel-based diffusion maps on OS and PFS.

Exploratory (translational) objectives are 1) to identify and quantify tumor-specific alterations in plasma DNA using NGS; 2) to characterize which of these tumor-specific alterations in plasma DNA form genomic and epigenetic determinants of tumor metabolic progression guided by FDG PET-CT; 3) to identify these tumor-specific alterations in previous tumor tissue; 4) to analyze whether CTC levels correlate with tumor metabolic progression guided by FDG PET-CT; and finally 5) to assess the prognostic value of CTCs on OS.

### Patient selection criteria

Baseline metabolic measurements for documentation of metabolic measurable disease by FDG PET-CT must be taken at study entry. Laboratory tests required for eligibility must be completed within 14 days prior to study entry.

### Inclusion criteria

Participants must have histologically confirmed CRC that is metastatic or unresectable and for which standard treatments do not exist or are no longer effective. In addition, patients should:

• be potential candidates for a Phase I study;

• have been treated with or be intolerant to all standard chemotherapeutic agents (fluoropyrimidines, irinotecan and oxaliplatin) and monoclonal antibodies (bevacizumab, cetuximab and/or panitumumab, regorafenib if available);

• have signed a written informed consent (approved by an Independent Ethics Committee [IEC]) and obtained prior to any study specific screening procedures;

• be aged 18 or older;

• have a life expectancy greater than 12 weeks;

• have an ECOG performance status ≤ 1;

• and show normal organ and marrow function as follows: total bilirubin within 2 × normal institutional upper limits, AST/ALT/Alk phosphatases levels < 5 × normal institutional upper limits, creatinine within 2 × normal institutional upper limits, or creatinine clearance > 35 mL/min.

• Women of child-bearing potential and men must agree to use adequate contraception (hormonal or barrier method of birth control, abstinence) prior to study entry and for the duration of study participation. Should a woman become pregnant or suspect she is pregnant while participating in this study, she must inform her treating physician immediately.

### Exclusion criteria

In addition to pregnant or breast-feeding women, excluded from the study are patients identified with any of the following conditions or characteristics:

• chemotherapy or radiotherapy within 4 weeks prior to entering the study or incomplete recovery from adverse events due to agents administered more than 4 weeks earlier.

• treatment with any experimental agents during the assessment time period.

• uncontrolled brain metastases.

• bleeding diathesis, history of cardiovascular ischemic disease, or cerebrovascular incident within the last six months.

• major surgery within four weeks.

• uncontrolled concurrent illness including, but not limited to, ongoing or active infection, symptomatic congestive heart failure, unstable angina pectoris, cardiac arrhythmia, psychiatric illness or any significant disease which, in the investigator’s opinion, would exclude the patient from the study.

• uncontrolled diabetes.

• a history of a different malignancy, except for the following circumstances: individuals with a history of other malignancies are eligible if they have been disease-free for at least 5 years and are deemed by the investigator to be at low risk for recurrence of that malignancy. Individuals with the following cancers are eligible if diagnosed and treated within the past 5 years: cervical cancer in situ, and basal cell or squamous cell carcinoma of the skin.

• contra-indications to the use of MRI: cardiac stimulator implanted cardiac wires, any implanted electronic devices, or intra-ocular metallic foreign bodies.

• a previous history of hypersensitivity to iodinated contrast media.

• medical, geographical, sociological, psychological or legal conditions that would not permit the patient to complete the study or sign informed consent.

### FDG-PET/CT imaging

Increased glycolysis is one of the hallmarks of cancer. FDG, an analogue of glucose labeled with a positron emitting isotope of Fluor (F^18^), is actively taken up in cancer cells of many tumor types. The positrons emitted by the FDG are detected by a dedicated camera, enabling the visualization of cellular glycolytic activity [37]. Serial FDG PET-CT consists of performing a scan at baseline (day 1) and after 2 weeks (day 15). The two PET-CTs need to be performed in strictly identical and standardized conditions.

The practical guidelines for FDG PET-CT imaging (activity injected; acquisition timing; processing; image analysis; PET-CT data form input) are specified in the Standard Procedure Imaging Manual (SPIM) for PET-CT, following as closely as possible the EANM procedure guidelines for tumor PET imaging [38]. Measurement of several FDG PET-CT metabolic parameters such as SUV, FTV and TLG for analysis will be documented. To respect FDG PET quantifications, an ultra-low dose CT (approx 1 mSv) will be performed to correct the metabolic images.

### Magnetic resonance imaging

The technical protocol will include T1 and T2 weighted images without contrast and a diffusion-weighted sequence with area under the curve calculation made on 2 B values with the first being superior to 150 ms to eliminate the fast component (microvessel-related) in order to get an expression of the true water diffusion properties of the tissue. The second B value will range between 800 and 1200 ms. The duration of this non-contrast imaging examination is about 20 minutes per patient. Volumetric, voxel-based vADC values will be computed with dedicated software at the sponsor institution (Institut Jules Bordet). ROI-based mean ADC value at the larger non-necrotic part of the lesion will also be determined.

### Genomic alterations

To detect tumor-specific alterations in plasma DNA via NGS technology, blood samples for plasma preparation will be collected at baseline (2 × 9 mL) and at 2 weeks (2 × 9 mL) after the start of the study (see Figure 1). An extra 9 mL whole blood sample will be collected at baseline in order to distinguish somatic from germline mutations. Extracted DNA samples will be used for further analysis using NGS. DNA will also be extracted from previously available tumor biopsies of the included patients in order to identify and quantify tumor-specific alterations.

### Circulating tumor cells

For CTC quantification, a 9 mL peripheral blood sample from each patient will be collected and sent at room temperature to the laboratory responsible for CTC detection at baseline and at 2 weeks after the start of the study (see Figure 1). These blood samples will be processed using Veridex, LLC,CellSearch®, and the identification and counting of CTCs will be performed with the CellSpotter™Analyzer, which is a semi-automated fluorescence-based microscopy system that permits computer-generated reconstruction of cellular images.

The laboratory investigators will be blinded to the clinical status of the patients.

### Follow-up

Follow-up procedures, performed every 2 months after the second PET-CT assessment, will include physical examination, vital signs and ECOG performance status, laboratory tests and diffusion-weighted MRI.

### Statistical considerations

Our primary analysis will consist of the assessment of the prognostic value of TMPI (evolution of the tumor FDG uptake from baseline to 2 weeks later) on OS. The patients will be divided into 2 groups using the observed median as threshold. The primary comparison will be done using Kaplan-Meier estimates of OS distributions and comparison using the log rank test (2-sided level of 5%). Based on published data from our team [19], we believe that a HR of .40 favoring patients with slow growing tumors could be expected and would have a clinically pertinent value. In order to detect such a HR if true, with a power of 80%, we need to have complete follow-up (observation until death) for 37 patients. Time zero for measuring survival will be the day of the second FDG PET-CT assessment.

Getting this number of events, assuming a median survival of 4 months for the overall population (i.e., we anticipate a median of 5.7 months for the patients with slow growing tumors and 2.3 months for the other patients), should be feasible with an accrual of 3 to 4 patients per month and registration of 47 patients with a FDG PET-CT evaluation after 2 weeks. An increase in sample size to 53 patients should compensate for the fact that not all patients will have a second FDG PET-CT assessment or at least one metabolic measurable lesion.

Analysis of the primary objective will be conducted using data from the patients who undergo the 2 FDG PET-CT evaluations.

## Ethical considerations

### Patient protection

The principal investigator ensures that this study conforms to the Declaration of Helsinki (available at http://www.wma.net/en/30publications/10policies/b3/) or the laws and regulations of the country, whichever provides the greatest protection of the patient.

The study follows the International Conference on Harmonization E 6 (R1) Guideline for Good Clinical Practice, reference number CPMP/ICH/135/95 (available at http://www.ich.org/fileadmin/Public_Web_Site/ICH_Products/Guidelines/Efficacy/E6_R1/Step4/E6_R1__Guideline.pdf).

The competent ethics committee of the Institut Jules Bordet approved the protocol, as required by applicable national legislation.

## Discussion

Tumor aggressiveness is one of the major determinants of patient outcome in advanced disease. Despite its importance, supported by findings reported in the literature of extreme outcomes for patients with mCRC treated with chemotherapy, no objective tool allows clinicians to base treatment decisions on this factor.

The CORIOLAN study will characterize TMPI using FDG-PET-based metabolic imaging of patients with chemorefractory mCRC during a period of time without treatment. Results will be correlated to other assessment tools like DW-MRI, CTCs and circulating DNA, with the aim to provide usable tools in daily practice and in clinical studies in the future.

## Abbreviations

ADC: Apparent diffusion coefficient; CTCs: Circulating tumor cells; DW-MRI: Diffusion-weighted magnetic resonance imaging; DWI: Diffusion-weighted imaging; EANM: European association of nuclear medicine; FDG-PET-CT: Fluoro deoxy glucose-positron emission tomography/computed tomography; FTV: Functional tumor volume; HR: Hazard ratio; mCRC: Metastatic colorectal cancer; MRI: Magnetic resonance imaging; NGS: Next generation sequencing; OS: Overall survival; PFS: Progression free survival; RECIST: Response evaluation criteria in solid tumor; ROI: Region of interest; SPIM: Standard procedures imaging manual; SUV: Standardized uptake value; TLG: Total lesion glycolysis; TMPI: Tumoral metabolic progression index; TTP: Time to progression; vADC: Volumetric evaluation of apparent diffusion coefficient.

## Competing interests

The authors report no conflicts of interest.

## Authors’ contributions

AD, PM, AH contribute to protocol writing, manuscript design, setting-up the trial, and writing manuscript; CV contributed to protocol writing, manuscript design and writing, and coordinate the translational research; CG and PF contribute to protocol writing, manuscript design, setting-up the trial, manuscript writing, and coordination of PET imaging network; ML and JLE contribute to protocol writing, manuscript design, setting-up the trial, manuscript writing, and coordination of MRI imaging; FH, PA, AA, NC, GM contribute to protocol writing, and setting-up the trial. All authors read and approved the final manuscript.

## Pre-publication history

The pre-publication history for this paper can be accessed here:

http://www.biomedcentral.com/1471-2407/14/385/prepub
